# Measurement of acute nonspecific low back pain perception in primary care physical therapy: reliability and validity of the brief illness perception questionnaire

**DOI:** 10.1186/1471-2474-14-53

**Published:** 2013-02-01

**Authors:** Joannes M Hallegraeff, Cees P van der Schans, Wim P Krijnen, Mathieu HG de Greef

**Affiliations:** 1 Hanze University of Applied Sciences, Groningen, the Netherlands; 2 University Medical Center Groningen, Department of Rehabilitation Medicine, Groningen, the Netherlands; 3 Institute for Master Education SOMT, Amersfoort, the Netherlands

**Keywords:** Illness perceptions, Reliability, Validity, Acute nonspecific low back pain, Brief IPQ

## Abstract

**Background:**

The eight-item Brief Illness Perception Questionnaire is used as a screening instrument in physical therapy to assess mental defeat in patients with acute low back pain, besides patient perception might determine the course and risk for chronic low back pain. However, the psychometric properties of the Brief Illness Perception Questionnaire in common musculoskeletal disorders like acute low back pain have not been adequately studied. Patients’ perceptions vary across different populations and affect coping styles. Thus, our aim was to determine the internal consistency, test-retest reliability and validity of the Dutch language version of the Brief Illness Perception Questionnaire in acute non-specific low back pain patients in primary care physical therapy.

**Methods:**

A non-experimental cross-sectional study with two measurements was performed. Eighty-four acute low back pain patients, in multidisciplinary health care center in Dutch primary care with a sample mean (SD) age of 42 (12) years, participated in the study. Internal consistency (Cronbach’s α) and test-retest procedures (Intraclass Correlation Coefficients and limits of agreement) were evaluated at a one-week interval. The concurrent validity of the Brief Illness Perception Questionnaire was examined by using the Mental Health Component of the Short Form 36 Health Survey.

**Results:**

The Cronbach’s α for internal consistency was 0.73 (95% CI, 0.67 – 0.83); and the Intraclass Correlation Coefficient test-retest reliability was acceptable: 0.72 (95% CI, 0.53 – 0.82), however, the limits of agreement were large. The Intraclass Correlation Coefficient measuring concurrent validity 0.65 (95% CI, 0.46 – 0.80).

**Conclusion:**

The Dutch version of the Brief Illness Perception Questionnaire is an appropriate instrument for measuring patients’ perceptions in acute low back pain patients, showing acceptable internal consistency and reliability. Concurrent validity is adequate, however, the instrument may be unsuitable for detecting changes in low back pain perception over time.

## Background

The natural course of recovery from acute nonspecific low back pain (ANLBP) is favorable, but recurrence within a year is high and primary care physiotherapy may be indicated [1,2]. Recurrence of ANLBP may be influenced by a patient’s behavior, as the cognitive and emotional process of pain often translates into complaints [3].

The multidimensional representations of the Common Sense Model (CSM) of self-regulation of illness reflect five cognitive dimensions: identity, consequences, cause, timeline, and cure or control [4-6]. In the context of ANLBP, these five areas characterize how low back pain patients view their disorder in terms of its cause, the condition itself, their expectations about recovery, and how to formulate their coping behavior [5,7]. Although illness perceptions of patients with common musculoskeletal disorders have not been studied adequately, it is generally recognized that patients’ illness perceptions do vary across different patient populations, and that illness perceptions can affect coping styles and the nature of subsequent complaints [8,9]. Indeed, psychological constructs, such as a patient’s perception of pain, determine the course and risk for chronic complaints of nonspecific low back pain [10]. Hence, a patient’s perception of ANLBP should be recognized as a potential risk factor for delayed recovery [11], one, in addition to the biomedical approach, that is potentially modifiable [12]. Indeed, there is evidence that treatment outcome for chronic low back, such as return to work, improve after changing patients’ illness perceptions [13]. This observation was corroborated by Hagger et al., who reported a relationship between perception of illness and mental health at several points in time [7].

These findings demonstrate the importance of assessing patients’ perceptions of their ANLBP, as their perceptions can influence their treatment and recovery. The Brief Illness Perception Questionnaire (IPQ-B) has been used widely in Dutch primary care for measuring the five cognitive representations of common musculoskeletal disorders, with the goal of altering patients’ perception towards their ANLBP. Broadbent et al. [14] derived the eight-item IPQ-B from the long 80-item version of the IPQ (IPQ-R) [15]. The IPQ-R is less suitable for use in daily clinical practice due to its length [15]. It is recommended to measure illness perceptions in terms of one psychological construct, one that can be measured repeatedly in a short period of time. The IPQ-B is a prime candidate for this purpose, as it quickly assesses cognitive perceptions of illness, such as consequences, timeline, personal control, treatment control, identity for describing the condition, the comprehensibility (coherence) of low back pain symptoms, concern, and emotions.

The psychometric properties of the IPQ-B have already been examined in a wide variety of illnesses and its correlation coefficients have been reported [14]. Moreover, the content validity of the IPQ-B and how its construct validity was measured in previous studies have undergone intensive discussion [16-18]. It is surprising that the psychometric properties of the Dutch version of the IPQ-B has never been assessed in ANLBP patients in primary care physiotherapy, given its widespread use in several musculoskeletal disorders in Dutch primary care physiotherapy and the importance of behavior in musculoskeletal disorders, in particular in ANLBP patients and their perception of pain. Therefore, the aim of this study was to assess the internal consistency, test-retest reliability, and concurrent validity of the Dutch version of the IPQ-B in primary care ANLBP patients.

## Methods

### Patients and setting

The study sample consisted of 84 patients with ANLBP consecutively recruited from three different primary care physical therapy providers localized in two medium-sized towns and one of them in rural areas in the northern part of the Netherlands. General practitioners screened all participants. Participants had a mean (SD) age of 42 (12) years; 43% were female.

Inclusion criteria: age 20–60 years, a new episode of acute non-specific low back (time since onset < 6 weeks) with or without radiating pain in the leg. Be capable to read and understand Dutch language.

Exclusion criteria: specific cause of low back pain like nerve root disorders, lumbar spinal stenosis, spondylolisthesis, after injury, infection, osteoporosis, tumour or rheumatic diseases such as M. Bechterew.

Before patients were included in the study, we obtained oral and written informed consent and explained to them the study protocol. Patient characteristics (gender, age, height, weight) and IPQ-B and SF-36 responses were obtained in a separate room prior to each patient’s scheduled standard care service. At this initial contact only anamnesis and physical examination were carried out after the data was collected. After one-week interval and before the second contact moment data of IPQ-B and SF-36 responses were again obtained. Physical therapists were instructed to avoid giving any information what might influence patients’ perception of low back pain. Intervention was carried out according to the Dutch guideline for ANLBP patients without controlling participants or conditions as every patient with ANLBP and as a result ethical approval was not required. All data were confidential to protect the health status of the participant and anonymity was guaranteed in electronic database. Ethics approval was not required because a purely observational, non-interactive study was carried out without interference in standard usual care and in accordance with normal practice and approvals. Research involving tests on cognitive, diagnostic or attitude procedures does not require ethics approval when data are completely and truly anonymous, participants can’t be identified, data will not cause any damage and participants consented to the use of the data. The study was performed in agreement with the directives given in the Helsinki Declaration as revised in 1975 [19]. Data collection was carried out from January 2011 to December 2011.

### Design

A non-experimental, cross-sectional study design was performed, with two measurement moments.

### IPQ-B

In 2006, Broadbent et al. constructed the briefer IPQ-B from the longer IPQ-R [14]. They assessed concurrent validity by examining correlations of items with the same construct [13,14]. The IPQ-B is an eight-item instrument that measures on an ordinal scale (0–10) a patient’s cognitive perceptions of his or her illness. Eight areas are examined: consequences (item 1), timeline (item 2), personal control (item 3), treatment control (item 4), identity for describing the condition and symptoms of low back pain (item 5), coherence (item 7), and concern and emotions (items 6 and 8). Items 3, 4, and 7 are reversed items. The maximum score on the IPQ-B is 80; higher scores reflect more negative perceptions of low back pain. To make the questionnaire suitable for ANLBP patients, we adapted the IPQ-B by replacing ‘illness’ with ‘low back pain’. De Raaij et al. developed a cross-cultural adaptation of the IPQ-B to make this scale applicable in the Netherlands. No minimal clinical difference or cut-off point is obtained [20,21].

### SF-36

The SF-36 health survey is an eight-scale generic and comprehensive instrument that measures physical and mental health and can be used in various musculoskeletal and medical disorders [22]. It has been intensively studied and validated for different musculoskeletal disorders, including low back pain [22,23]. Its psychometric quality is high. The higher the score on the SF-36, the better the status of health. In this study, we used the ‘acute form’ of the SF-36. There is no gold standard measure for the assessment of concurrent validity of the IPQ-B. Broadbent et al. 2008 stated that use of mental health care is high when illness perceptions are more negative [24]. In contrast with a disease-specific health survey this generic health survey can be used across ages with several disorders and treatment groups.

We examined the correlation of the IPQ-B with the Mental Health Component score (MCS) of the SF 36 consisting of the domains “mental health”, “role-emotional”, “social functioning” and “vitality”. The SF 36 MCS is useful to compare correlations with other instruments measuring the same construct. Besides, this measure makes it possible to compare results across different populations such as acute nonspecific low back pain patients.

Cronbach‘s alpha coefficient of the MCS summed score 0.76.

### Statistical analyses

We used SPSS 19.0. For normally distributed data, patient characteristics and descriptive statistics were presented as means and standard deviations. Internal consistency was assessed using Cronbach’s α analysis and confidence interval (95%). We assessed the test-retest reliability of the IPQ-B after a one-week interval to measure the same entities at two different time points and calculated intraclass correlation coefficients (ICCs) and confidence interval (95%). An ICC value above 0.75 is indicative of good reliability, whereas values below 0.75 are indicative of moderate reliability [23].

Pearson’s correlation coefficient was used to assess concurrent validity with the MCS of the SF-36.

We used a Bland Altman plot to show the Limits Of Agreement (LOA) between two measurements on a ratio scale: the mean values of the test and retest assessments and mean difference between the two assessments, considering 95% of the results vary between the mean difference. LOAs are indicators of agreement and the plot is for visual judgment reflecting the relationship between the mean and the difference of the two measurements. LOA can be considered to be an assessment of measurement error. The time interval of the test-retest measurements is a random effect in the model.

## Results

### Patients’ characteristics

Twenty-one subjects were excluded from this study due to either lost to follow-up or chronic lower back complaints due to nerve root disorders, rheumatic diseases, or other specific causes. A total of 84 patients participated in this study and completed the first assessment successfully. The data were normally distributed, as determined using the Kolmogorov-Smirnov test (P > 0.05), and showed no floor or ceiling effects (< 15% or > 15% of the highest or lowest score). Table 1 shows the patients’ characteristics.

**Table 1:** Patient characteristics

**Total number of patients**	**84**
Age (years), mean (SD)	42 (12)
Female, n (%)	36 (43%)
Relapse^a^ – yes	28
Sports^b^ – yes	40
Education^c^	
Low	10
Intermediate	45
High	29
Height (cm), mean (SD)	178 (9)
Weight (kg), mean (SD)	81 (15)
BMI (kg/m^2^), mean (SD)	25 (4)
Pain (millimeter), mean (SD)	57 (20)

^a^Last previous episode was < 6 months.

^b^Organised sports.

^c^Low = Primary school; intermediate = secondary education; high = higher education.

### Internal consistency

The inter-item consistency of the IPQ-B was 0.73 (Cronbach’s α; 95% CI, 0.67 – 0.83).

Table 2 shows internal reliability of the IPQ-B.

**Table 2:** Items of the IPQ-B and internal reliability

**Items of the IPQ-B**	**Scale means**	**Cronbach’s α**
	**if item deleted**	**if item deleted**
Consequences	46	0.65
Timeline	45	0.72
Personal control	46	0.68
Treatment control	44	0.73
Identity	47	0.69
Concern	45	0.66
Coherence	45	0.70
Emotional response	45	0.63

### Test-retest reliability

There was a significant difference between the first assessment and second assessment (t=−3.5 [*P* < 0.05]). This reduces the reliability of the IPQ-B, with a mean difference of 4.1 (95% CI, -6.4 – -1.7). Test-retest over a one-week period showed an acceptable correlation, measured by an ICC of 0.72 (95% CI, 0.53 – 0.82) two-way random effects model, absolute agreement.

**Figure 1:**
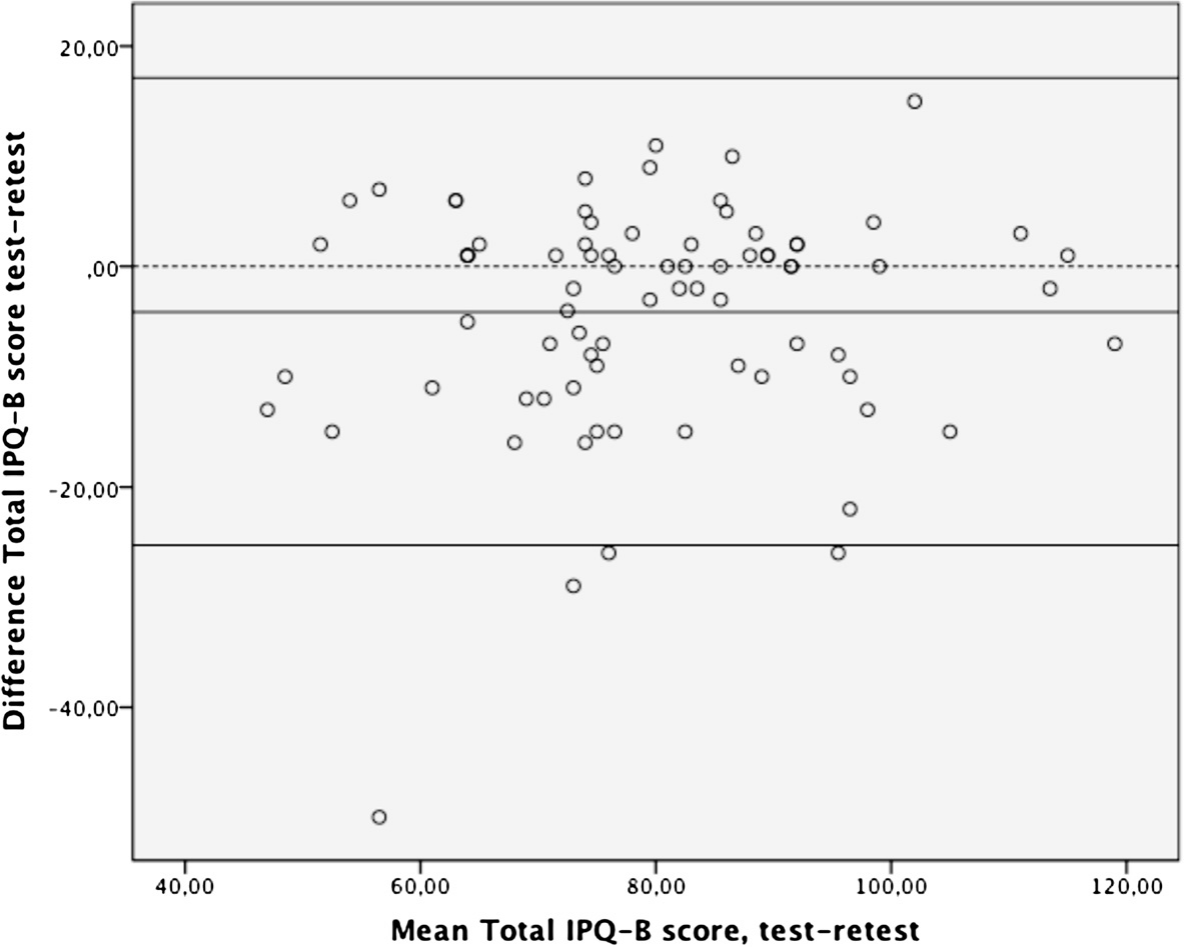
**Bland Altman plot for the test-retest reliability of the IPQ-B.** A total of 84 patients participated in the test-retest assessment. The dotted line represents no difference between test and retest. The central line representing the mean difference between test and retest scores, which was - 4.1, and the 95% limits of agreement are presented as flanking lines.

The 95% upper and lower LOA was 21.2 (− 4.1 ± 21.2) LOA ranged from −25.3 to 17.1 as shown in the Bland Altman plot (Figure 1). This indicates that the variance of the repeated measurements for each subject is independent of the mean of the repeated measurements. No systematic trend was visible. The standard error of the mean (SEM) was 1.17, and the smallest detectable change (SDC) was 42. Thus, a change in the IPQ-B score must exceed a value of 42 in order to reflect a true difference between test and retest scores.

### Concurrent validity

When compared with the mental health component scale (MCS) of the SF-36, concurrent validity of the IPQ-B had an ICC of 0.65 (95% CI, 0.46 – 0.80). The Pearson’s correlation coefficient was *r*_p =_ 0.51, (*P* < 0.01), indicating adequate validity between the two instruments. Correlation coefficients of the four separate scales of the Mental Health component of the SF-36 with the IPQ-B showed: Vitality: *r*_p =_ 0.54^**^; Social Functioning *r*_p =_ 0.45^**^; Role Emotional *r*_p =_ 0.43^**^; Mental Health *r*_p_ = 0.59^**^. **Correlation is significant at the 0.01level (two-tailed) (Table 3).

**Table 3:** Pearson’s correlations between IPQ-B items and the mental health component of the SF-36

	**Mental health component SF-36**
IPQ-B	0.51^**^
Consequences	0.49^**^
Timeline	0.15
Personal control	0.35^**^
Treatment control	0.28^*^
Identity	0.23
Concern	0.48^**^
Coherence	0.28^*^
Emotional response	0.53^**^

*Correlation is significant at the 0.05 level (two-tailed). **Correlation is significant at the 0.01 level (two-tailed).

## Discussion

This is the first study to evaluate the Dutch version of the IPQ-B in ANLBP patients. The internal reliability, test-retest reliability, and concurrent validity indicate that the Dutch IPQ-B is of moderate psychometric quality. Even though the IPQ-B measures a psychological construct by means of a multidimensional scale with a few items, we found its internal consistency of 0.73 to be adequate [25]. None of the items will affect the overall reliability if they were deleted. Kline, as well as other authors, agrees that an internal consistency of ≥ 0.60 indicates sufficient reliability for psychological constructs [26]. According Terwee et al., an acceptable test-retest reliability must exceed an ICC value of > 0.70 [27]. Although the lower limits of the 95% CI in the present study was less than 0.60, the ICC was an acceptable 0.72 (95% CI, 0.53 – 0.82). For concurrent validity, no gold standard is available for assessing patients’ perception of acute low back pain. Therefore, we used the criteria of Nunnally et al. [28] to determine that in our study the concurrent validity of the IPQ-B with the MCS of the SF-36 to be adequate.

In contrast to the ICC values, which demonstrated adequate test-retest reliability, the LOAs in the Bland Altman plot were large. The large LOAs might have been due to fewer low back complaints over time resulting from intervention-related changes in the patients’ perception of their back pain. Participants reported more positive perceptions on the IPQ-B retest than test (t – 3.5, *P* < 0.05). Therefore, it was preferable to shorten the test-retest interval time in the study. Most ANLBP decreases within the first weeks after onset, and as a result, negative perceptions concerning ANLBP also decrease [1]. To mitigate this phenomenon as much as possible, we instructed the examiners in the primary care units that had contact with the patients to avoid giving patients any information about the course of ANLBP that could influence their perception of pain. As a consequence of the positive natural course of ANLBP recovery, patients’ perception might also have been influenced, especially during the acute stage.

However, the maximum score of the IPQ-B is 80. In the present study, the SDC was 42, which means that a change in IPQ-B score must exceed a value of 42 in order to reflect a true difference between test and retest scores; random error also explains the decrease of IPQ-B score. An SDC value of 42 also indicates that there is low agreement between the two scores, and thus moderate longitudinal responsiveness to real changed perception of complaints. We conclude, therefore, that the instrument is not suitable for detecting real individual changes.

For concurrent validity, Terwee et al. proposed a correlation value of ≥ 0.50 to be acceptable [27]. In our study, the Pearson’s correlation coefficient was 0.51 and the ICC value for the IPQ-B and the mental health subscale of the SF-36 was 0.65 (95% CI, 0.46-0.80). However, since the items of the IPQ-B are derived from earlier versions of the IPQ, the content validity of the scale might have been influenced during this derivation process. The IPQ-B was developed by ‘forming one question that best summarized the items contained in each subscale of the IPQ-R’ [13]. Indeed, more recent findings indicate that people do have difficulties understanding the items of the IPQ-B, with some even misinterpreting them [15]. This could influence the content validity of the instrument, leading to the question: ‘Is the scale really measuring the same construct?’ [15]. Nonetheless, in the present study, we did find the internal consistency of the scale to be adequate in ANLBP patients.

The results of our study are consistent with those reporting on the psychometric properties of the IPQ-B for several illnesses. However, our findings differ from those of Broadbent et al., who also used the mental health component of the SF-36 to determine concurrent validity in myocardial patients [13]. They found negative associations for four items of the IPQ-B when compared to the mental health subscale. A possible explanation for this disparity is that psychological state has a greater impact on ANLBP patients than on patients with a specific medical condition such as myocardial infarction.

Small sample size was a major limitation of the study; results must be interpreted with caution. Another limitation was the relatively long test-retest period, so patients could have been influenced by the favourable natural course and a positive change in pain and activities might have occurred. These developments changed the perception of low back pain that might have negatively biased the test-retest reliability results. One problem inherent of this kind is to minimize treatment influence; hence, all data was collected just before the two interventions. However, an explanation of the changed IPQ-B score might be that internal and/or external influences between both administrations have affected patients’ perceptions of low back pain.

Main and George emphasized the importance of measuring a patient’s perception as part of a more psychologically informed physical therapy practice [11]. The goal of doing so is to identify and alter the patient’s perception of musculoskeletal pain and response to pain in his or her daily coping behavior, as a patient’s cognition of his or her pain and disability might be essential for decreasing musculoskeletal disorders and for a more rapid recovery [11]. Therefore, we emphasize the need for measuring patient pain perception for several musculoskeletal disorders. At the same time, we need to acknowledge the complexity of this multilevel representation and the problems patients might have interpreting the items of instruments measuring this psychological construct. In this regard, we support the use of the IPQ-B in primary care physical therapy management, as it is a useful instrument to assess patients’ *initial* perceptions of their disorder. Such assessments should address more negative perceptions of patients’ back pain, with the aim of decreasing the risk of more chronic low back pain problems.

## Conclusions

On the basis of the data from this study, we conclude that the IPQ-B is a suitable instrument for measuring patients’ perception in acute nonspecific low back pain patients however this measure needs further examination with another criterion measure and with a larger patient population. The instrument may be unsuitable for detecting changes in low back pain perception over time.

## Competing interests

No reimbursements, fees, funding or salary have been received that may gain or lose financially from the publication of this manuscript. Such an organization has not financed this manuscript. Authors do not hold or applying for any patents relating to the content of the manuscript and no reimbursements, fees, funding or salary have been received from an organization that holds or has applied for patents relating to the content of the manuscript. No stocks, shares or patents are hold by the authors relating to the content of the manuscript that may gain or lose financially from the publication of this manuscript. No other financial or non-financial (political, personal, religious, ideological, academic, intellectual, commercial or any other) competing interest in relation to this manuscript is present.

## Authors’ contributions

JH initiated the study and was responsible for the acquisition of data. All authors contributed substantially to the study including the conception and design (JH, MG, CS), analysis and interpretation of data (JH, WK) drafting the article or revising it critically for important intellectual content. All authors read and approved the final manuscript.

## Pre-publication history

The pre-publication history for this paper can be accessed here:

http://www.biomedcentral.com/1471-2474/14/53/prepub
